# Unraveling the mysteries of dog evolution

**DOI:** 10.1186/1741-7007-8-20

**Published:** 2010-03-09

**Authors:** Rodney L Honeycutt

**Affiliations:** 1 Department of Biology, National Science Division, Pepperdine University, 24255 Pacific Coast Highway, Malibu, California 90263-4321, USA

## Abstract

The increased battery of molecular markers, derived from comparative genomics, is aiding our understanding of the genetics of domestication. The recent *BMC Biology *article pertaining to the evolution of small size in dogs is an example of how such methods can be used to study the origin and diversification of the domestic dog. We are still challenged, however, to appreciate the genetic mechanisms responsible for the phenotypic diversity seen in 'our best friend'.

## Background

Size and shape are the hallmarks of the mammalian radiations and these two features are emblematic of the remarkable diversity of families, orders and genera of mammals that vary widely in form, yet share a common ancestry. The ordinal-level diversity of mammals is especially noteworthy, reflecting conformational changes in the skull, dentition and postcranial skeleton that result in forms as divergent as bats and whales. From a paleontological standpoint, diversification of many orders occurred over a relatively short period of time [1], which makes changes in form even more curious. One potential model for understanding the genetic basis of evolutionary change in mammalian form is the domestic dog, *Canis familiaris*. Domestication and strong directional selection (for example, artificial selection) for phenotypic and behavioural traits have resulted in morphological diversity within the domestic dog unparalleled in any wild mammalian species. In approximately 15,000 years the level of morphological divergence among dog breeds has exceeded that seen between many genera of wild canids [2-4]. Today, the 400 plus breeds of dogs vary in size, shape of the skull and modifications of the postcranial skeleton (in particular the limb bones) to a degree that would suggest species-level, if not generic-level, differences, if their remains were discovered in the wild.

Darwin's [5] entire theory of evolution by means of natural selection provides a material explanation for diversity of form in nature. He used examples from domesticated plants and animals as analogies for how adaptation can arise from selection acting on variations that cause differences in reproductive success across generations. As Gregory [6] indicates, we can learn many lessons about evolution in natural systems through detailed studies of our domesticated species, and variation in the domestic dog raises a number of questions commonly asked about wild species of mammals: (1) what is the domestic dog's closest relative?; (2) where did the domestic dog originate and was there a single origin or multiple origins of domestic dog lineages?; (3) when did domestication of the dog occur?; (4) what genetic changes accompany the differences observed among breeds of dogs and their wild canid ancestors?

As with the studies of human origins over the past two decades [7-10], many of the above questions related to the domestic dog are being addressed in considerable detail with the use of phylogenetics, population genetics, molecular biology and comparative genomics [11-19]. This commentary was prompted by a recent paper in *BMC Biology *[20] that addressed differences in size among breeds of dogs as well as the timing and origin of small-sized dogs. The foundation for this paper originated with the work of Sutter *et al. *[21], who used a genome-wide survey and association analysis in the Portuguese water dog to identify a QTL (quantitative trait locus) on chromosome 15 that sorted with body size. In particular, *IGF1 *(insulin-like growth factor 1) was suggested as a candidate gene for body size variation in domestic dogs and variation at 116 SNPs (single nucleotide polymorphisms) for 526 dogs clustered into two major groups, small and large breeds. Gray *et al. *[20] expanded upon these findings by using specific molecular markers (SNPs, microsatellite loci, insertion/deletion of a SINE element and nucleotide sequences) to characterize the segregation of domestic dogs into two major groups, small and large body size. As shown by their study, most small breeds of dog have two unique markers (SINE element insertion in intron 2 of the *IGF1 *gene and a SNP allele) not found in either wolves or large breeds. Based on the phylogenetic and geographic distribution of sequence variants associated with the 'small dog haplotype', these authors concluded that small dogs originated in the Middle East, as they share a relationship with wolves from this region. Furthermore, they suggest that changes unique to small size occurred early in the evolution of domestic dogs. As a result of these findings and more recent molecular-based studies on the evolution of the domestic dog, I will provide an update on how close we are to resolving the above questions.

## Ancestry of the domestic dog

**Figure 1:**
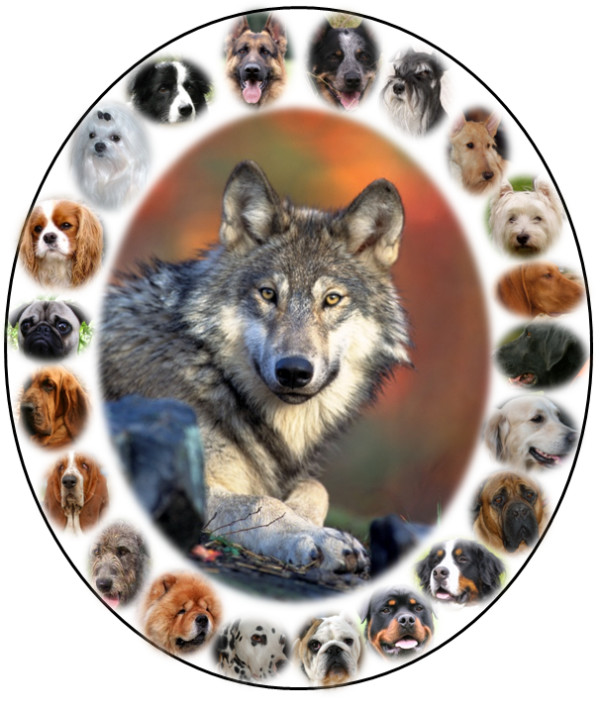
**The wolf's family portrait reveals a diversity of form among breeds of domestic dogs**. Images for the figure are from Wikipedia. The Cavalier King Charles Spaniel was posted by Ellen Levy Finch, the Bassett, Dalmatian, Mastiff, and Scottish Terrier by Lilly M, and the Vizsla by Briantresp. The gray wolf photograph was taken by Gary Kramer, U.S. Fish and Wildlife Service.

Darwin [5] stated that 'I do not believe, as we shall presently see, that all our dogs have descended from any one wild species'. Rather, he suggested that domestic dogs 'descended from several wild species'. Phylogenetic analyses derived from molecular markers support an origin of the domestic dog from one ancestor, the wolf (*Canis lupus*), thus refuting Darwin's hypothesis ([11,22]; Figure 1). The unresolved issue relates to whether or not all lineages of dogs originated from a single wolf stock or multiple stocks of wolves. Most studies of variation at the mitochondrial control region suggest that patterns of relationship among dog and wolf mitochondrial lineages is the result of multiple origins of dogs from different wolf stocks followed by introgressive hybridization between dogs and wolves [11,22,23]. A recent study of variation at the Mhc (major histocompatibility) locus also suggested that the high level of variation observed at this locus is best explained by continued backcrossing between dogs and wolves subsequent to domestication [16]. These results contrast with a recent study [19] based on mitochondrial DNA (mtDNA) that implies an origin for the domestic dog from a 'single gene pool', rather than multiple domestication events and continued hybridization with wolf stocks. There is one reason why I doubt the conclusions from Pang *et al. *[19]. Their analysis compared patterns of variation in 1576 dog mtDNA to 40 wolf sequences. Such asymmetry in sampling of the wolf population is likely to bias any conclusions about origin. Given the fact that wolves, dogs and other members of the genus *Canis *are inter-fertile [24-26], there is a high likelihood that dogs and wolves interbred subsequent to hybridization, thus complicating the derivation of the number of founders for dog lineages.

## Time and centre of the origin of the domestic dog

Hypotheses related to the geographic and temporal origin of the domestic dog are reminiscent of studies related to human origins. One of the more practical obstacles that must be overcome relates to the conflicts between estimates of time since divergence based on archaeology and those derived from a molecular clock. Based on a calibration point between wolf and coyote and a mitochondrial molecular clock, time since divergence between the wolf and domestic dog was estimated to be between76, 000 and 135,000 years ago [11], which is considerably higher than the 13,000 to 17,000 years ago based on archaeological evidence [27]. This discrepancy is not resolved by more recent interpretations of molecular data and, in many cases, the basis for a reassessment of molecular data is not clear. For instance, Gray *et al. *[20] indicated that small dogs originated about 12,000 years ago, yet they fail to indicate how they had arrived at this date. It appears that their argument is based primarily on archaeological evidence that reveals the first appearance of a small dog phenotype in the Middle East. Nevertheless, older dates for the origin of dogs have been reported and one must question a date based on archaeology alone, especially since information from archaeology has been used to support several different centres of origin for the domestic dog. Pang *et al. *[19] also suggested that the domestic dog originated less than 16,300 years ago, but details for the molecular calibrations are lacking. As a result of conflicts between dates derived from molecular and archeological data, it appears that most recent molecular studies embrace data provided by archaeological evidence. The discrepancy seen between divergence times derived from fossil materials and molecules is not unique to dog origins. Part of the incongruence relates to the inherent error associated with any estimates of time since divergence for recent divergences among lineages, especially when the origin of such lineages is complicated by the possibility of multiple origins from an ancestral stock and admixture [17].

Issues related to estimates for the centre of origin for the domestic dog are still complicated and, again, it relates to how one interprets the archaeological and molecular data. As reviewed by Verginelli *et al. *[23], some of the earliest fossils identified as dog occur between 12,000 and 17,000 years ago in Europe and the Middle East, and there is some evidence for Eastern European wolf populations contributing to the origin of the domestic dog. In contrast, based on higher levels of mtDNA variation in dogs from East Asia and the general phylogeographic partitioning of that variation, Savolainen *et al. *[28] argue for a single East Asian origin of the domestic dog and this conclusion appears congruent with some archaeological evidence [29].

The problem with this particular study, however, relates to the small number of wolf samples examined relative to dogs. Given the diversity in wolf populations distributed worldwide, one would think that a large number of individuals and populations of the ancestral species should be examined. The arguments by Gray *et al. *[20] for the origin of small-sized dogs in the Middle East are based on the similarity between wolves from the Middle East and small dogs. Nevertheless, from a phylogenetic standpoint, support for this hypothesis is tenuous, given the small bootstrap values. As an alternative, one might argue that the two shared traits associated with small size in dogs may reflect convergence as a result of artificial selection for size rather than divergence from a single common ancestor in the Middle East.

## Genetic basis of morphological diversity in the domestic dog

Despite the high level of phenotypic variation among breeds, genetic divergence within the domestic dog and between most species of the genus *Canis *is quite low. All species of *Canis *have identical karyotypes [30] and genetic comparisons based on mitochondrial and nuclear genes reveal low levels of divergence between members of this genus [11,22,31]. In part, this level of genetic similarity explains the level of inter-fertility seen among species of *Canis*. As suggested by Gray *et al. *[32], the dog experienced two population bottlenecks, the first associated with domestication and the second with the formation of various breeds, with the latter responsible for most of the loss in genetic diversity. This has resulted in much higher linkage disequilibrium in dogs compared to humans [13]. Although mtDNA markers fail to reveal breed-specific markers [11], both microsatellite loci [33] and SNPs [15] are capable of assigning individual purebred dogs to their specified breed. Nevertheless, genetic markers to date are considerably less effective at providing well-supported phylogenetic groups of breeds, primarily as a result of most breeds differing more by allele frequency than fixed differences. Therefore, reconstructing the overall phylogeny of domestic dogs is considerably more complicated as a result of the recent origin of many breeds coupled with high levels of admixture during breed formation.

Deciphering the underlying genetic causes of morphological diversity in the domestic dog presents considerable challenges. Top-down approaches [34], using a QTL mapping, linkage disequilibrium mapping and association analyses are all methods that take advantage of the dog genome sequence. Furthermore, such methods bypass the need for large pedigrees. Such an approach has proven useful in identifying candidate genes and the mutations responsible for traits associated with spotting and the hair ridge in Rhodesian ridgebacks [18]. These same methods allowed Sutter *et al. *[21] and Gray *et al. *[20] to identify a chromosomal region whose variation appears to be associated with size differences in dogs. Despite these advances as a result of comparative genomics and marker-assisted mapping, deciphering the mechanisms responsible for the origin of form in the domestic dog will be challenging. For instance, the QTL identified by Sutter *et al. *[21] appears to be associated with size, yet variation at the *IGF1 *locus does not appear to be 'a major contributor to body size in all small dogs'. Association analyses are an excellent first approximation but multifactorial traits resulting from gene/environment interactions and epistasis complicate our understanding of the genetic basis of form.

As stated by Carroll [35], 'The key to understanding form is development'. The question still remains - What processes are responsible for the diversity of forms observed in the domestic dog? Rather than major modifications in structural genes, changes may be considerably more subtle and involve changes in the timing of gene expression, the alteration of interactions among various gene products and variation in regions of genes controlling development. Such changes might allow for changes in the phenotype without major genetic divergence. The domestic dog may very well offer clues to the types of changes in form observed in nature, such as those observed for the mammalian radiations, and this is the reason why continued research on genes controlling development in dogs is an exciting avenue of research.

## Abbreviations

GF: insulin growth factor; mtDNA: mitochondrial DNA; QTL: quantitative trait locus; SNP: nucleotide polymorphisms.
